# Factors associated with functional dependence in older adults with disabilities in Brazil: a study based on the 2019 *National Health Survey*

**DOI:** 10.1590/0102-311XEN208525

**Published:** 2026-09-18

**Authors:** Dalia Elena Romero, Leo Ramos Maia, Paulo Roberto Borges de Souza

**Affiliations:** 1 Instituto de Comunicação e Informação Científica e Tecnológica em Saúde, Fundação Oswaldo Cruz, Fiocruz, Rio de Janeiro, Brasil.

**Keywords:** Aged, Health Inequiquities, Persons with Disabilities, Personal Autonomy, Prevalence Studies, Anciano, Inequidades en Salud, Personas con Discapacidad, Autonomía Personal, Estudios de Prevalencia

## Abstract

This study aimed to analyze factors associated with moderate and severe functional dependence in older adults with sensory or motor disabilities in Brazil. Data from the 2019 *Brazilian National Health Survey* were used in this cross-sectional study. The sample included 9,856 older adults (aged ≥ 60 years) with hearing, visual, or motor impairment. Functional dependence was chosen as the outcome variable, classified into three levels: independence, moderate functional dependence, and severe functional dependence. Personal and environmental factors were considered in this study. Crude and adjusted prevalence estimates and multinomial logistic regression models were estimated, adopting independence as the reference category. Among the older adults with disabilities, 44.4% lived independently, 33.6% had moderate dependence, and 22% experienced severe dependence. Severe or moderate functional dependence occurred more often in women, older individuals, and those with less schooling. Race/skin-color was only associated with severe functional dependence , with lower odds in Mixed-race individuals than in White ones. Regarding the environmental factors, higher chances of dependence occurred in those who occupied no leadership position in their household, residents of the Brazilian Northeast, and users of assistive technologies. Co-residency, receiving home visits from community health workers, and using regular rehabilitation care were specifically associated with severe functional dependence. The high proportion of older adults with disabilities who live independently challenges ableist conceptions about aging. However, the observed inequalities indicate that social and environmental factors still conditioned the maintenance of functional autonomy.

## Resumen

El objetivo de este estudio fue analizar los factores asociados con la dependencia funcional (moderada y severa) entre adultos mayores con discapacidades sensoriales o motoras en Brasil. Este es un estudio transversal que utiliza datos de la *Encuesta Nacional de Salud* del 2019. La muestra incluyó a 9.856 adultos mayores (≥ 60 años) con discapacidad auditiva, visual o motora. La variable de desenlace fue la dependencia funcional, clasificada en tres niveles: independencia, dependencia funcional moderada y dependencia funcional severa. Se tuvieron en cuenta factores personales y factores ambientales. Se calcularon estimaciones de prevalencia y modelos de regresión logística multinomial brutos y ajustados, adoptando la independencia como categoría de referencia.Entre los adultos mayores con discapacidades, el 44,4% vivía de forma independiente, el 33,6% tenía una dependencia moderada y el 22% tenía una dependencia severa.La dependencia funcional (severa o moderada) fue más frecuente entre las mujeres, las personas mayores y aquellas con menor nivel educativo. La raza/color se asoció únicamente con la dependencia funcional severa, con probabilidades más bajas entre las personas pardas en comparación con las blancas. Entre los factores ambientales, se observaron mayores probabilidades de dependencia entre quienes no ocupaban un puesto de liderazgo en el hogar, los residentes de la Región Nordeste y los usuarios de tecnologías asistivas. La corresidencia, las visitas domiciliarias de agentes comunitarios de salud y el uso de cuidados periódicos de rehabilitación se asociaron específicamente con la dependencia funcional severa. La elevada proporción de personas mayores con discapacidad que viven de forma independiente desafía las concepciones capacitistas sobre el envejecimiento. Sin embargo, las desigualdades observadas indican que el mantenimiento de la autonomía funcional sigue estando condicionado por factores sociales y ambientales.

## Introduction

Within the “successful aging” paradigm, autonomy and independence are pillars established in the literature as key indicators of well-being ^1^ ^,^ ^2^. These pillars emerge, among other reasons, to overcome exclusionary biases and bring classical aging paradigms closer to reality. On the one hand, the excessive focus on preserving bodily functions and avoiding disability that inform these classical paradigms tends to become ableist by assigning lower value to people with disabilities ^2^. On the other, belief that a significant portion of the population can reach advanced age without experiencing any disability is not supported by population studies ^1^ ^,^ ^2^.

According to data from the 2022 *Demographic Census* ^3^ in Brazil, 45% of peoploe with disabilities (approximately 6.5 million) are 60 years of age or older, representing one-fifth of the country’s older population. Among the oldest-old, those aged 80 or older, 40% live with some form of disability. Moreover, given the rapid population aging seen in Brazil, the share of older adults with disabilities is expected to increase ^4^. In this context, valuing old age with independence − recognizing that a good life is possible even with disabilities − proves to be a more realistic and less ableist goal for Brazilian society than the narrow prioritization of aging without disability ^2^.

Autonomy, a central element for the well-being of older adults with disabilities, can be defined as the capacity to maintain a sense of personal agency, self-efficacy, and the power of choice ^2^. Wehmeyer ^5^, in turn, highlights self-determination as an essential element of autonomy, understood as an individual’s ability to exercise control over their own lives. From this perspective, functional independence − understood as the absence of a need for assistance from others to perform daily activities ^6^ − constitutes an important aspect to be considered regarding the health of this population.

Neither functional independence nor self-determination is a matter of individual will or ability, but rather a result of empowering people with disabilities ^5^. Empowering, in turn, means ensuring the means, knowledge, and opportunities to make self-determination possible.

From an empowerment perspective, the environment is the most crucial dimension, as it enables or restricts the autonomy of people with disabilities ^5^. This dimension encompasses both tangible aspects − such as physical infrastructure and rehabilitation services − and intangible ones − such as attitudes, cultures, and beliefs ^7^.

The *International Classification of Functioning, Disability and Health* (ICF) constitutes the main framework used to operationalize the different aspects of functioning, including the interactions between environmental factors and individual characteristics ^7^. In this model, functional capacity refers to an individual’s ability to perform activities and participate in social life, resulting from the interaction between their health conditions and the context in which they live. Functional independence, in turn, is lost from the moment this capacity is reduced to the point of compromising autonomy to perform daily activities.

Interactions between the environment and the living conditions of people with disabilities is reflected in the unequal prevalence of independence across territories and social groups ^8^. Such consequences ^7^, in turn, tend to be more pronounced in the older population due to the cumulative impact of social determinants of health over the course of life ^9^.

Research in Brazil has evinced social inequalities among older people regarding functional independence ^10^. Overall, the most affected groups are the oldest-old, women, and those with lower socioeconomic status. Factors associated with functional status, however, vary according to the degree of dependence ^11^.

However, studies on dependency among older people in Brazil focus on the general older population ^6^ ^,^ ^10^ ^,^ ^12^. Thus, the factors associated with dependency among older adults with disabilities remain unknown, even though autonomy is a core aspect for understanding the well-being of people with disabilities ^2^.

Based on the above scenario, we elaborated two research hypotheses: (1) that older adults with disabilities can live independently in Brazil; (2) that personal and environmental factors mediate the conditions for independent living in this population, which would manifest as inequalities regarding dependency prevalence.

Thus, this study analyzed factors associated with moderate and severe functional dependence among older adults with sensory or motor impairment in Brazil.

## Methods

### Design and data source

This cross-sectional, analytical, population-based study included a representative sample of the population living in private households in Brazil ^13^. The 2019 *Brazilian National Health Survey* (PNS, acronym in Portuguese) ^14^, conducted by the Brazilian Institute of Geography and Statistics (IBGE, acronym in Portuguese) between August 2019 and March 2020, was used as the data source.

PNS is a household-based survey with a complex sample design. Its 2019 edition included 94,114 interviewed households. To ensure sample representativeness for the Brazilian population, expansion factors were applied, including a correction factor for non-response. Details on the sampling method, weighting factors, and data collection are available in previous papers ^13^ ^,^ ^14^.

The 2019 PNS questionnaire consisted of three parts. The first two contain data on all household members (n = 279,372), including housing characteristics (section 1); socioeconomic conditions and access to and use of health services; and information on disabilities and the health status of individuals over 60 and under 2 years of age (section 2). The third section contains information about one randomly selected resident from each household and addresses issues related to health, lifestyle, social support, and work characteristics.

Only the first two sections were used in the present study, aiming at a larger sample.

### Sample and variables

Sample inclusion criteria consisted of being 60 years of age or older and having some type of disability. Individuals who self-identified as Indigenous (n = 128) or Asian (n = 82) were excluded due to the low number of cases, as were those with an intellectual disability (n = 1,339), since the question used to identify this condition already incorporates reports of functional difficulty in activities of daily living, which would render the analysis tautological. The final sample consisted of 9,856 older adults with sensory or motor disabilities.

Classified as older adults with sensory or motor disabilities were those who, on a permanent basis, experienced great difficulty or were completely unable to perform at least one of the following activities: seeing; hearing; walking or climbing stairs; lifting a two-liter bottle of water from waist to eye level; and picking up small objects, such as buttons and pencils, or opening and closing containers or bottles.

Functional status was chosen as the outcome variable, classified into three levels: independence, moderate functional dependence, and severe functional dependence.

Individuals who required assistance to perform activities of daily living (ADL) were considered to have severe functional dependence. They were identified based on a “Yes” response to the question: “Do/Does need help performing any of these activities (eating, bathing, using the toilet, dressing, walking from one room to another in the house, getting in or out of bed unassisted, or sitting down or standing up from a chair unassisted)?”

Individuals were classified as having moderate functional dependence if they required assistance with instrumental activities of daily living (IADL) but not with ADLs. Requiring assistance with IADLs was identified based on a “Yes” response to the question: “Do/Does need help performing any of these activities (shopping, managing finances, taking medication, going to the doctor, going out using transportation − bus, subway, taxi, car, etc.)?”

Individuals who did not possess moderate functional dependence and severe functional dependence were classified as functional independence.

Based on the ICF’s ^7^ definition of functioning, this study included the following independent variables:

(i) Personal factors: (1) gender (male and female); (2) age group in years (60-69; 70-79; and 80 or older); (3) race/ethnicity (White, Black, or Mixed-race); (4) schooling level (illiterate, incomplete primary education, complete primary and secondary education, incomplete tertiary education or higher); (5) per capita household income in minimum wages (up to 1/2, more than 1/2 up to 1, more than 1). 

(ii) Household-related environmental factors: (1) older adults with sensory or motor disabilitie co-residence status (living alone, living with one person, or living with two or more people); (2) household status (head of household/spouse of the head or other household status); (3) macro-region of residence; (4) adequate housing − considered adequate if the dwelling simultaneously met the following criteria: resident-to-bedroom density ≥ 2, piped water in at least one room, sewage disposal via a general sewer network, storm drain system, or septic tank, and garbage collection by a sanitation service ^15^; (5) internet access in the home. 

(iii) Environmental factors related to the use of health technologies and services: (1) frequency of home visits by community health workers (CHW) in the last 12 months; (2) use of assistive technology for their disability, considering individuals who used assistive technology/technologies designed to aid the impaired function(s); (3) receipt of regular rehabilitation care in the last 12 months (such as physical therapy, occupational therapy, speech therapy, psychotherapy, etc.). 

### Statistical analysis

Percentage distribution of older adults with sensory or motor disabilities according to environmental factors and the prevalence of each dependency level were estimated considering the survey’s sampling design, along with the respective 95% confidence intervals (95%CI).

Factors associated with functional dependence in older adults with sensory or motor disabilities were investigated by multinomial logistic regression models in which the dependent variable − functional dependence − considered three categories: independence (reference), moderate and severe. Crude analyses were followed by adjusted models, which included only those independent variables showing a statistically significant association in the crude analysis. Model results were presented as odds ratios (OR), along with their respective confidence intervals and p-values, using a 5% significance level.

All analyses were performed using the IBM SPSS (https://www.ibm.com/) statistics software package (version 21) via the complex samples module.

The 2019 PNS project was approved by the Brazilian National Research Ethics Committee, Brazilian Nationa Health Council (CONEP/CNS; Opinion n. 3,529,376).

## Results

In 2019, nearly half (44.4%; 95%CI: 42.8-46.0) of older adults with sensory or motor disabilities residing in private households in Brazil were independent (Table 1), 33.6% (95%CI: 32.0-35.1) had moderate functional dependence, and 22% (95%CI: 20.7-23.4) had severe functional dependence.

Regarding the older adults with sensory or motor disabilities sociodemographic profile (Table 1), over 60% were female and aged 70 years or older, and slightly more than half were Black (Black or Mixed-race). Precarious living conditions in this population are striking: 78% had not completed primary education, and only 37% had a per capita household income equal to or greater than 1 minimum wage. 

**Table 1:** Degree of functional status and personal and environmental characteristics among older adults (aged 60 years or older) with sensory or motor impairment. Brazil, 2019.

Variables	n	%	95%CI
Total	9,856	100.0	-
Functional status			
Severe dependence	2,170	22.0	20.7-23.4
Moderate dependence	3,308	33.6	32.0-35.1
Independence	4,377	44.4	42.8-46.0
Personal Factors			
Gender			
Male	3,435	34.9	33.5-36.2
Female	6,420	65.1	63.8-66.5
Age group (years)			
60-69	3,950	40.1	38.5-41.7
70-79	3,286	33.3	31.9-34.9
80 or older	2,619	26.6	25.1-28.1
Race/Ethnicity			
White	4,677	47.5	45.8-49.1
Black	1,132	11.5	10.4-12.6
Mixed-race	4,047	41.1	39.6-42.6
Per capita household income (minimum wages)			
Up to 1/2	1,777	18.0	16.8-19.3
1/2 to 1	4,501	45.7	44.0-47.4
Over 1	3,576	36.3	34.6-38.0
Schooling level			
Illiterate/No formal education	2,654	26.9	25.5-28.4
Incomplete primary education	5,047	51.2	49.6-52.8
Secondary education	1,654	16.8	15.6-18.0
Incomplete tertiary education or over	500	5.1	4.4-5.8
Environmental factors			
Internet access at home			
Yes	5,722	58.1	56.3-59.8
No	4,133	41.9	40.2-43.7
Co-residency			
Lives alone	1,949	19.8	18.5-21.1
Lives with one person	3,829	38.8	37.2-40.5
Lives with two people or more	4,077	41.4	39.8-43.0
Household status			
Head or spouse	8,148	82.7	81.4-83.8
Neither head nor spouse	1,708	17.3	16.2-18.6
Lives in adequate housing *			
No	2,816	28.6	27.0-30.2
Yes	7,040	71.4	69.8-73.0
Region of residence			
North	681	6.9	6.5-7.4
Northeast	2,931	29.7	28.6-30.9
Southeast	4,241	43.0	41.6-44.5
South	1,424	14.5	13.7-15.3
Central-West	578	5.9	5.4-6.3
Frequency of CHW home visits			
Monthly	2,867	29.1	27.5-30.7
From 1 to 6 visits	2,420	24.6	23.1-26.0
No visits	4,569	46.4	44.5-48.2
Received rehabilitation care in the last 12 months			
Yes	2,056	20.9	19.6-22.2
No	7,800	79.1	77.8-80.4
Makes use of assistive technology			
No	5,972	60.6	59.1-62.1
Yes	3,883	39.4	37.9-40.9

95%CI: 95% confidence interval; CHW: community health workers.

Source: 2019 *Brazilian National Survey of Health* ^14^.

* Adequate housing: up to 2 residents per bedroom, piped water, adequate sanitary sewage and garbage collection.

Both older adults with sensory or motor disabilities living alone and those who did not hold a leadership role in the household reached almost 20% (Table 1). About 7 out of 10 resided in the Southeast or Northeast regions, a proportion similar to that of households with adequate housing. Conversely, fewer homes had internet access (42% did not have it).

Most older adults with sensory or motor disabilities did not use assistive technologies for their disability (61%) and had not receive CHW home visits in the 12 months prior to the survey (46%) (Table 1). 21% of the sample received regular rehabilitation care during the same period. However, the type of care received is unknown, and it may not be related to the rehabilitation of the impaired bodily function.

Table 2 shows the degree of functional dependence among the older adults with sensory or motor disabilities, according to personal and environmental characteristics. Severe functional dependence was more prevalent among those who were not the heads/spouses of the head of household (43.9%; 95%CI: 40.0-47.8), aged 80 years and over (37.2%; 95%CI: 34.3-40.2), who used assistive technology (31.9%; 95%CI: 29.5-34.5), and who received regular rehabilitation care in the last year (31.6%; 95%CI: 28.5-34.9). Those aged 60-69 years, who lived alone and with incomplete tertiary education represented the least affected, showing a severe functional dependence prevalence below 15%.

**Table 2:** Degree of functional status among elder adults with sensory or motor impairment according to personal and environmental characteristics. Brazil, 2019.

Variables	Degree of functional dependence for activities of daily living								
	Independent			Moderate dependence			Severe dependence		
	n	%	95%CI	n	%	95%CI	n	%	95%CI
Total	4,377	44.4	42.8-46.0	3,308	33.6	32.0-35.1	2,170	22.0	20.7-23.4
Personal factors									
Gender									
Male	1,791	52.1	49.5-54.7	906	26,4	24.2-28.7	738	21,5	19.3-23.9
Female	2,586	40.3	38.3-42.3	2,402	37.4	35.4-39.4	1,432	22.3	20.7-24.0
Age group (years)									
60-69	2,427	61.4	59.0-63.9	1,001	25.4	23.2-27.6	522	13.2	11.5-15.2
70-79	1,442	43.9	41.4-46.4	1,170	35.6	33.1-38.2	674	20.5	18.4-22.8
80 or older	508	19.4	16.8-22.3	1,137	43.4	40.4-46.5	974	37.2	34.3-40.2
Race/Ethnicity									
Mixed-race	1,831	45.3	43.0-47.5	1,392	34.4	32.1-36.7	823	20.3	18.6-22.2
Black	496	43.9	39.2-48.7	386	34.1	30.0-38.4	249	22.0	18.3-26.2
White	2,049	43.8	41.3-46.3	1,530	32.7	30.4-35.1	1,098	23.5	21.4-25.7
Schooling level									
Incomplete tertiary education or over	333	66.7	60.1-72.7	92	18.4	14.0-23.8	75	14.9	10.8-20.2
Secondary education	897	54.2	50.4-58.0	394	23.8	20.9-26.9	364	22.0	18.9-25.4
Primary education	2,307	45.7	43.5-47.9	1,709	33.9	31.7-36.0	1,031	20.4	18.7-22.3
Illiterate/No formal education	840	31.6	29.0-34.5	1,114	42.0	39.1-44.9	700	26.4	23.9-29.1
Per capita household income (minimum wages)									
Up to 1/2	776	43.7	40.0-47.4	598	33.6	30.2-37.3	403	22.7	19.8-25.8
Over 1/2 up to 1	1,875	41.7	39.4-43.9	1,625	36.1	34.0-38.2	1,001	22.2	20.4-24.2
Over 1	1,725	48.2	45.6-50.9	1,085	30.3	27.7-33.1	766	21.4	19.3-23.8
Environmental factors									
Internet access at home									
No access	1,722	41.7	39.3-44.1	1,551	37.5	35.2-39.9	860	20.8	18.8-23.0
Have access	2,655	46.4	44.3-48.5	1,757	30.7	28.8-32.7	1,310	22.9	21.1-24.7
Household status									
Head or spouse of the head	4,036	49.5	47.8-51.3	2,690	33.0	31.4-34.7	1,421	17.4	16.1-18.9
Other role	341	20.0	17.1-23.1	618	36.2	32.6-39.9	749	43.9	40.0-47.8
Co-residency									
Lives alone	945	48.5	44.7-52.3	751	38.5	35.0-42.2	253	13.0	10.8-15.5
Lives with one person	1,770	46.2	43.6-48.9	1,233	32.2	29.9-34.6	826	21.6	19.3-24.1
Lives with two people or more	1,662	40.8	38.4-43.1	1,323	32.5	30.2-34.8	1,092	26.8	24.8-28.9
Region of residence									
North	305	44.7	41.0-48.5	248	36.5	32.7-40.5	128	18.8	16.1-21.8
Southeast	1,885	44.4	41.6-47.3	1,479	34.9	32.0-37.8	877	20.7	18.4-23.1
Central-West	266	45.9	41.1-50.9	184	31.8	27.8-36.1	129	22.3	18.4-26.8
South	776	54.5	50.9-58.0	329	23.1	20.3-26.1	319	22.4	19.6-25.5
Northeast	1,146	39.1	36.6-41.7	1,068	36.4	34.0-38.9	717	24.5	22.1-27.0
Adequate housing *									
Yes	3,222	45.8	43.8-47.8	2,306	32.8	30.9-34.7	1,512	21.5	20.0-23.1
No	1,155	41.0	38.5-43.6	1,002	35.6	33.0-38.3	658	23.4	20.8-26.2
CHW home visits in the year									
Monthly	2,187	47.9	45.5-50.3	1,456	31.9	29.7-34.1	925	20.2	18.3-22.3
From 1 to 6 visits	1,016	42.0	39.1-44.9	878	36.3	33.3-39.3	526	21.7	19.5-24.1
No visits	1,174	40.9	37.9-44.1	974	34.0	31.0-37.1	719	25.1	22.6-27.7
Regular rehabilitation in the year									
Received no care	3,621	46.4	44.6-48.2	2,659	34.1	32.3-35.9	1,520	19.5	18.0-21.1
Received care	756	36.8	33.5-40.1	649	31.6	28.5-34.8	651	31.6	28.5-34.9
Assistive technology									
Does not use	2.964	49.6	47.6-51.7	2,079	34.8	32.9-36.7	930	15.6	14.2-17.1
Uses	1,414	36.4	33.9-39.0	1,230	31.7	29.2-34.2	1,240	31.9	29.5-34.5

95%CI: 95% confidence interval; CHW: community health workers.

Source: 2019 *Brazilian National Survey of Health* ^14^.

* Adequate housing: up to two residents per bedroom, piped water, adequate sanitary sewage and garbage collection.

Moderate functional dependence, in turn, was more prevalent among those aged 80 years or older (43.4%; 95%CI: 40.4-46.5), were illiterate (42%; 95%CI: 39.1-44.9), and lived in single-person households (38.5%; 95%CI: 35.0-42.2) (Table 2). Lowest prevalence (< 25%) was observed among older adults with sensory or motor disabilities with higher schooling levels and those residing in the South Region.

Finally, older adults with sensory or motor disabilities who were younger, had higher schooling levels, resided in the South region, were male, and did not use assistive technology presented higher functional independence prevalence (Table 2).

Logistic regression models with moderate functional dependence as the outcome and independent older adults with sensory or motor disabilities as the reference category revealed that both personal and contextual factors influence the likelihood of exhibiting this level of dependency (Table 3). Of the personal factors, age group and schooling showed a strong association with moderate functional dependence in the adjusted analysis. Odds of presenting moderate functional dependence was 4.45 times higher (95%CI: 3.50-5.64) among people aged 80 years and over compared with those aged 60-69 years. Similarly, illiterate individuals were 3.33 times more likely (95%CI: 2.22-5.01) to have moderate functional dependence than those with incomplete tertiary education or higher. Females were also associated with a higher moderate functional dependence prevalence, presenting 88% more chances (95%CI: 1.60-2.21) compared with men. Although per capita household income showed an association in the crude analysis, it lost statistical significance after adjustment for the other factors included in the model.

**Table 3:** Crude and adjusted analysis of factors associated with moderate functional dependence in older adults with sensory or motor impairment. Brazil, 2019.

Variables	Moderate dependence				
	Crude OR			Adjusted OR		
	OR	95%CI	p-value	OR	95%CI	p-value
Personal factors						
Gender						
Female	1.84	1.58-2.14	< 0.001	1.88	1.60-2.21	< 0.001
Age group (years)						
80 or older	5.42	4.35-6.77	< 0.001	4.45	3.50-5.64	< 0.001
70-79	1.97	1.67-2.32	< 0.001	1.80	1.52-2.14	< 0.001
60-69	1.00	-	-	1.00	-	-
Race/Ethnicity						
Mixed-race	1.02	0.87-1.20	0.824	-	-	-
Black	1.04	0.82-1.32	0.733	-	-	-
White	1.00	-	-	-	-	-
Schooling level						
Illiterate/No formal education	4.82	3.35-6.94	< 0.001	3.33	2.22-5.01	< 0.001
Incomplete primary education	2,69	1.89-3.82	< 0.001	2.40	1.65-3.49	< 0.001
Secondary education	1.59	1.09-2.34	0.017	1.61	1.08-2.40	0.020
Incomplete tertiary education or over	1.00	-	-	1.00	-	-
Per capita household income (minimum wages)						
Up to 1/2	1.23	0.98-1.53	0.075	1.14	0.89-1.46	0.305
Over 1/2 up to 1	1.38	1.17-1.63	< 0.001	1.13	0.93-1.36	0.212
Over 1	1.00	-	-	1	-	-
Environmental factors						
Internet access at home						
No access	1.36	1.18-1.57	< 0.001	1.12	0.95-1.33	0.186
Co-residency						
Lives with two people or more	1.00	0.82-1.22	0.987	-	-	-
Lives with one person	0.88	0.72-1.07	0.199	-	-	-
Lives alone	1	-	-	-	-	-
Household status						
Neither head nor spouse	2.72	2.19-3.38	< 0.001	1.96	1.55-2.48	< 0.001
Adequate housing						
No	1.21	1.04-1.41	0.012	1.07	0.90-1.26	0.456
Region of residence						
North	1.92	1.50-2.46	< 0.001	1.73	1.33-2.25	< 0.001
Northeast	2.20	1.78-2.70	< 0.001	1.87	1.49-2.35	< 0.001
Southeast	1.85	1.48-2.31	< 0.001	1.85	1.47-2.32	< 0.001
Central-West	1.63	1.24-2.14	< 0.001	1.49	1.12-1.99	0.006
South	1.00	-	-	1	-	-
CHW home visit in the last 12 months						
Received at least 1 visit	1.27	1.09-1.47	0.002	1.15	0.97-1.36	0.101
Regular rehabilitation care in the past 12 months						
Yes	1.17	0.97-1.41	0.094	-	-	-
Assistive technology						
Uses	1.24	1.06-1.45	0.007	1.27	1.08-1.49	0.004

95%CI: 95% confidence interval; CHW: community health workers; OR: odds ratio.

Source: 2019 *Brazilian National Survey of Health* ^14^.

Notable among the contextual factors that remained associated with moderate functional dependence in the adjusted analysis were household role, region of residence, and the use of assistive technology (Table 3). Older adults who were neither heads nor spouses of the head of household were 1.96 (95%CI: 1.55-2.48) times more likely to present moderate functional dependence compared with those in these roles. Higher odds were observed among residents in the Northeast (OR = 1.87; 95%CI: 1.49-2.35), Southeast (OR = 1.85; 95%CI: 1.47-2.32), North (OR = 1.73; 95%CI: 1.33-2.25) and Central-West (OR = 1.49; 95%CI: 1.12-1.99) compared with South ones. Less significant, the use of assistive technology was also associated with moderate functional dependence, with those reporting its use being 27% (95%CI: 1.08-1.49) more likely to present moderate functional dependence.

As for the models that presented severe functional dependence as an outcome (Table 4), we observed a relative similarity among the factors associated with moderate functional dependence. Among personal factors, age group showed the strongest association, with the odds of severe functional dependence being 7.00 times higher (95%CI: 5.39-9.10) among individuals aged 80 years or older compared with those aged 60-69 years. Schooling level also presented a strong association, with higher odds among individuals with lower educational level: illiterate individuals were 3.14 (95%CI: 1.93-5.12) times more likely to have severe functional dependence compared with those who had incomplete tertiary education or higher. As for gender, females had 27% more odds of presenting severe functional dependence than men (OR = 1.27; 95%CI: 1,05-1,53). Unlike the analysis of the moderate functional deendence outcome regarding race/ethnicity, Mixed-race individuals had 22% lower odds (OR = 0.78; 95%CI: 0.62-0.98) of presenting severe functional dependence compared with White ones. Per capita household income did not maintain a statistically significant association after adjustment for the other variables included in the model, similar to the moderate functional dependence model.

**Table 4:** Crude and adjusted analysis of factors associated with severe functional dependence in older adults with sensory or motor impairment. Brazil, 2019.

Variables	Severe dependence				
	Crude OR			Adjusted OR		
	OR	95%CI	p-value	OR	95%CI	p-value
Personal factors						
Gender						
Female	1.34	1.13-1.59	0.001	1.27	1.05-1.53	0.014
Age group (years)						
80 or older	8.91	7.00-11.34	< 0.001	7.00	5.39-9.10	< 0.001
70-79	2.17	1.75-2.71	< 0.001	2.12	1.67-2.68	< 0.001
60-69	1.00	-	-	1.00	-	-
Race/Ethnicity						
Mixed-race	0.84	0.71-1.00	0.044	0.78	0.62-0.98	0.034
Black	0.94	0.70-1.25	0.662	0.93	0.66-1.31	0.68
White	1.00	-	-	1.00	-	-
Schooling level						
Illiterate/No formal education	3.73	2.47-5.62	< 0.001	3.14	1.93-5.12	< 0.001
Incomplete primary education	2.00	1.35-2.96	0.001	2.21	1.42-3.44	< 0.001
Secondary education	1.81	1.19-2.77	0.006	2.20	1.37-3.53	0.001
Incomplete tertiary education or over	1.00	-	-	1.00	-	-
Per capita household income (minimum wages)						
Up to 1/2	1.17	0.93-1.47	0.181	1.36	1.00-1.84	0.052
Over 1/2 up to 1	1.20	1.01-1.43	0.037	1.24	1.00-1.55	0.051
Over 1	1.00	-	-	1.00	-	-
Environmental factors						
Internet access at home						
No access	1.01	0.85-1.21	0.888	-	-	-
Co-residency						
Lives with two people or more	2.46	1.92-3.14	< 0.001	1.48	1.13-1.93	0.005
Lives with one person	1.75	1.33-2.29	< 0.001	1.60	1.20-2.14	0.001
Lives alone	1.00	-	-	1.00	-	-
Household status						
Neither head nor spouse	6.24	4.97-7.83	< 0.001	3.92	3.01-5.11	< 0.001
Adequate housing *						
No	1.21	1.01-1.46	0.042	1.13	0.91-1.41	0.276
Region of residence						
North	1.02	0.78-1.34	0.887	1.20	0.87-1.65	0.279
Northeast	1.52	1.20-1.92	< 0.001	1.56	1.16-2.10	0.004
Southeast	1.13	0.89-1.43	0.307	1.19	0.90-1.57	0.22
Central-West	1.18	0.85-1.63	0.319	1.18	0.83-1.68	0.371
South	1.00	-	-	1.00	-	-
CHW home visit in the last 12 months						
Received at least 1 visit	1.34	1.14-1.58	< 0.001	1.27	1.04-1.54	0.019
Regular rehabilitation care in the past 12 months						
Yes	2.05	1.68-2.50	< 0.001	2.85	2.26-3.60	< 0.001
Assistive technology						
Uses	2.80	2.34-3.33	< 0.001	2.76	2.27-3.36	< 0.001

95%CI: 95% confidence interval; CHW: community health workers; OR: odds ratio.

Source: 2019 *Brazilian National Survey of Health* ^14^.

* Adequate housing: up to two residents per bedroom, piped water, adequate sanitary sewage and garbage collection.

Regarding environmental factors, the adjusted analysis revealed a significant association between severe functional dependence and variables related to household arrangements, region of residence, and access to health services and resources (Table 4). Household status showed the strongest association, with the odds of severe functional dependence being 3.92 times higher among older adults with sensory dependence disabilities who were neither the head of the household nor the spouse of the head (95%CI: 3.01-5.11). Regarding co-residence, a higher likelihood of severe functional dependence was observed among those living with two or more people (OR = 1.48; 95%CI: 1.13-1.93) or with only one person (OR = 1.60; 95%CI: 1.20-2.14) compared with those living alone. Region of residence also showed a significant association, with 56% higher odds of severe functional dependence among residents of the Northeast Region (OR = 1.56; 95%CI: 1.16-2.10) compared with residents of the South Region. 

Individuals with access to health services and technologies were more likely to present severe functional dependence (Table 4). Receiving a home visit from a CHW was associated with 27% higher odds of severe functional dependence (OR = 1.27; 95%CI: 1.04-1.54). However, stronger associations were observed regarding the use of regular rehabilitation care (OR = 2.85; 95%CI: 2.26-3.60) and assistive technology (OR = 2.76; 95%CI: 2.27-3.36), showing higher odds of severe functional dependence among those who reported using these resources.

## Discussion

A first notable finding in the present study, capable of demystifying ableist biases present in various paradigms on health and aging, is that almost half of older adults with sensory or motor disabilities in Brazil lead functionally independent lives. Although a descriptive and simple estimate, it alone confirms our first research hypothesis that life with disabilities can occur independently, even for older adults.

However, the possibilities of experiencing this independence are unequal, as evinced by the analysis of factors associated with functional dependence. Inequalities observed between the analyzed groups suggest that both socially constructed barriers (material and immaterial) and biological factors influence the conditions for independent living among this population. Functional dependence occurred more often in women, older, and those with less schooling, evincing important sociodemographic inequalities. Regarding environmental factors, older adults with sensory or motor disabilities who occupy no leadership role in the household, live in the Brazilian Northeast, and use assistive technologies presented higher chances of experiencing functional dependence, both moderate and severe. Co-residency, receiving home visits from CHWs and regular rehabilitation care were specifically associated with severe functional dependence.

Particular characteristics of the factors associated with each degree of dependence in the older adults with sensory or motor disabilities population are consistent with studies on the general older adults population ^11^. However, common aspects between both outcomes included per capita household income, internet access, and adequate housing, which were not significantly associated with neither outcome in the adjusted models.

Women presented disadvantages in relation to the prevalence of functional dependence, especially in the moderate degree. These findings disagree with nationwide ^12^ and local ^11^ ^,^ ^16^ studies focusing on the general older population. In the first case ^12^, impairment to perform IADLs was associated with female gender, albeit with a lower frequency than that observed here, with odds 26% higher than those of men. Conversely, no association was observed for ADLs ^12^. Such differences indicate that disadvantages related to functional status in the older adult population are accentuated for disabled women. Local studies found no association between gender and the analyzed outcomes ^11^ ^,^ ^16^. Biological factors related to the higher prevalence of disabling diseases among women ^17^ are often cited to explain female disadvantages regarding functional status in the older adults population ^16^.

Advanced age was the main factor associated with loss of independence among older adults with sensory or motor disabilities. Despite strong association with both degrees of dependence, it was prominently marked for severe functional dependence, with significantly higher odds among those over 80 years of age. Association between age and dependence, marked by biological processes that generate a decline in functioning and are intrinsic to individual aging, has also been observed in studies on the general older adults population ^11^ ^,^ ^12^ ^,^ ^16^. In these studies, however, the association between functional disability and age group was less prominent and stronger for IADLs than for ADLs ^11^ ^,^ ^16^. Such differences indicate that for adults with disabilities over 80, maintaining independence becomes a particularly challenging condition.

Race/ethnicity showed no significant association with moderate functional dependence among older adults with sensory or motor disabilities, consistent with a study focused on the general older adults population in Brazil ^12^. However, Mixed-race individuals had 22% lower odds of severe functional dependence compared with White ones. Considering the social, cultural, and phenotypic heterogeneity that characterizes the “Mixed-race” category in Brazil, formulating interpretations ^18^ about the factors that could explain this difference is difficult.

Socioeconomic inequality in the degree of dependence among older adults with sensory or motor disabilities was observed by verifying schooling level. Disadvantages emerged for those with less schooling, in both moderate and severe dependence. Given that disability among older adults in Brazil is strongly associated with schooling level, being 2.23 times more prevalent among illiterate older adults compared with those reporting incomplete tertiary education or higher4, this reality proves to be even more problematic. This means a double burden of disadvantages for less educated older individuals: they acquire disabilities more frequently and, once impaired, are more likely to lose their independence.

Such association between functioning and schooling results from a multiplicity of aspects that mediate the relation between health status and the level of formal education ^19^. One aspect mentioned in the literature concerns the greater understanding and adoption of healthy habits by better educated people, which contributes to preserve their functional capacity ^20^. In this regard, engaging in physical activities throughout life ^21^ is extremely important among older adults with sensory or motor disabilities to develop and preserve muscle mass, thereby preventing sarcopenia ^22^. Often, this condition leads to the development and worsening of motor disabilities − the most prevalent type of disability in the older adults population ^4^.

Less access to and worse use of health services and technologies by people with less schooling are also frequently mentioned issues ^21^. Low educational level subjects individuals to a lower knowledge, acceptability and understanding of health techniques and technologies for management and functional rehabilitation capable of preventing or postponing dependence due to their disability. Interpersonal and institutional discrimination resulting from a triple stigmatization of these people as: (i) older adults, (ii) illiterate, and (iii) disabled ^23^, may also affect their accessibility to such techniques and technologies.

Working unqualified jobs throughout the life course ^24^ may also explain the lower prevalence of independence among less educated older adults with sensory or motor disabilities. Older adults with less schooling more often worked degrading jobs that have deteriorated their physical conditions for years ^25^.

Finally, the association between schooling and moderate functional dependence was slightly greater than with severe functional dependence, consistent with studies on the general older adult population ^12^ ^,^ ^16^. As IADLs involve greater complexity, older adults with less schooling are expected to request help for performing them more frequently than for basic tasks. In extreme cases, notably frequent in older adults population, illiteracy makes it impossible or extremely difficult to independently carry out activities such as going to the doctor, shopping, managing finances and taking medication ^16^.

Unlike schooling, household income was not associated with dependence, a result consistent with a study conducted in Montes Claros, Minas Gerais State, Brazil ^26^. One possible interpretation is that the material conditions to access services and goods that preserve or manage functioning have little influence on the independence status of older adults with sensory or motor disabilities. Another possible explanation is the success of Social Assistance and Social Security policies, which reduced inequalities and made the poverty level in the older population residual ^27^.

Notably, in many cases the association between environmental factors and functional dependence occurred unexpectedly. Internet access at home, which could facilitate IADL ^28^, is an example. One possible interpretation is that internet usage by older adults with sensory or motor disabilities remains limited and yields little benefit, regardless of access.

Another example was adequate housing. By including access to sanitary sewage and piped water as an adequacy criterion ^15^, this indicator was expected to be associated with older adults with sensory or motor disabilities’s independence to perform ADLs, especially hygiene-related ones. Housing structure is fundamental for people with disabilities to live independently ^7^. A simple example is that of someone with a lower-limb impairment living in a home where the use of stairs is necessary. In this case, dependency on help to leave the house becomes inevitable. As the questions focused on households in the PNS do not include characteristics about adaptability for disabled people, adopting a more appropriate criterion of adequate housing was unfeasible.

Receiving rehabilitation care and using assistive technologies also showed unexpected results. In assuming that: (i) the entire sample, as a result of their disabilities, would require rehabilitation care and use assistive technologies; (ii) the use of these resources might preserve autonomy ^29^, we expected a lower prevalence of dependence among its users.

These findings reflect the limitation of cross-sectional research, which does not allow for establishing cause-and-effect relations. Most likely, the search for rehabilitation and assistive technologies is driven by the severe degree of functional disability ^30^. More solid evidence about assumption “ the use of these resources might preserve autonomy” requires, at least, longitudinal studies with independent older adults with sensory or motor disabilities.

Regarding the less frequent use of these resources by older adults with sensory or motor disabilities with functional dependence or severe functional dependence, two explanations are possible: (i) low acceptability of people with greater functional capacity toward assistive technologies due to the stigma attached to their use ^31^; (ii) barriers to access, if the public offer is insufficient and prioritized for worst-condition individuals. In this scenario, we must question whether the late use of rehabilitation services and assistive technologies by older adults with sensory or motor disabilities with functional independence or moderate functional dependence would not be leading to avoidable or early cases of severe functional dependence.

Uncertainty regarding the cause-and-effect relation also applies to associations with CHW home visits. Although home care favors monitoring, preserving and rehabilitating the functioning of older adults with sensory or motor disabilities ^32^, the association found indicates that primary health care (PHC) prioritizes severe functional dependence individuals. Given their difficulties in commuting to healthcare facilities, such prioritization is partly justified.

Conversely, the associations related to the family environment were as expected. However, these associations also seem to be caused by dependency, whereas dependency compels older adults with sensory or motor disabilities to undergo a family rearrangement; it is not, therefore, a condition generated by the family environment. Given their need for help with basic daily activities, co-residing with the dependent older adults makes life easier for family caregivers ^33^. Concurrently, the moment older adults with sensory or motor disabilities become care-dependent, they tend to lose their leadership role in the home environment ^34^.

Finally, one of the most significant inequalities in terms of needing public health services was related to the region of residence. Older adults with sensory or motor disabilities living in the Northeast, Southeast, North and Central-West regions had higher chances of presenting moderate functional dependence than those living in the South. Notably, residing in the Northeast yielded greater odds for moderate functional dependence and an association with severe functional dependence. A study using the 2013 PNS found a similar scenario for the general older population, with greater disadvantages in the Northeast ^35^. Such a significant association between regional inequality and moderate functional dependence is understandable since IADLs are more related to the out-of-home environment. We can therefore infer that the South offers better conditions for older adults with sensory or motor disabilities to live independently. More comprehensive studies on the determinants of this inequality would be valuable for guiding policies aimed at overcoming these regional inequalities, fostering urban policies to adapt public and private spaces and services in less inclusive territories.

Other policies aimed at promoting the independence of people with disabilities and reducing the inequalities observed should include: (i) PHC strengthening by expanding and qualifying the provision of rehabilitation care and health promotion actions, thereby contributing to preserve the functional capacity of poeople with disabilities ^36^; (ii) the provision of appropriate assistive technologies for managing functional status in people with disabilities, associated with monitoring and guidance that ensure appropriate use ^20^; and (iii) the adaptation of households to the specific needs of each individual, favoring greater autonomy and security in daily life ^7^.

The COVID-19 pandemic has highlighted the urgency and magnitude of the problem of caring for dependent older adults. In the most critical moments of the health crisis, many older adults with functional dependence were deprived of basic care or received it from often overburdened family members ^37^. Since health crisis situations are expected to increase due to climate change ^38^, society must prepare for this scenario via actions on multiple fronts, including the prevention and delay of functional dependence.

Some study limitations, like the cross-sectional research design, have already been discussed throughout the study. Another important limitation concerns the possible survivorship bias ^39^. older adults with sensory or motor disabilities with worse living conditions may not even reach dependence or survive for a short time after reaching it. Consequently, the magnitude of the inequalities observed tends to be undersized in relation to the real severity.

On the other hand, this study has important strengths. As a unprecedented national investigation, it utilizes a representative sample of a group that, although numerous, has been little studied, thereby addressing a critical information gap on people with disabilities.

Knowledge regarding the care dependency status of people with disabilities is especially relevant to emerging care policies. A key milestone of this agenda, the recently approved National Care Policy (*Law n. 15,069/2024*) ^40^ defines “*people with disabilities who require assistance, support, or aid to perform basic and instrumental activities of daily living*” as one of its priority target groups. Currently, it is not possible to determine the profile and size of this group at the national level among non-older adults populations. Given this context, we underscore the urgency of including questions about requiring help to perform ADLs and IADLs for all peolpe with disabilities in future surveys.

## Data Availability

The sources of information used in the study are indicated in the body of the article.
