# Supplementary material for: Factors associated with functional dependence in older adults with disabilities in Brazil: a study based on the 2019 National Health Survey
Source: Cad Saude Publica. 2026 Sep 18;42:e00208525. doi: 10.1590/0102-311XEN208525 (PMC13592778; doi:10.1590/0102-311XEN208525)
Supplement: Translation

# Translation: Fatores associados à dependência funcional em pessoas idosas com deficiência no Brasil: estudo com base na *Pesquisa Nacional de Saúde* de 2019

**DOI:** 10.1590/0102-311XPT208525

**Authors:** Dalia Elena Romero, Leo Ramos Maia, Paulo Roberto Borges de Souza

**Affiliations:** 1 Instituto de Comunicação e Informação Científica e Tecnológica em Saúde, Fundação Oswaldo Cruz, Fiocruz, Rio de Janeiro, Brasil.

**Keywords:** Idoso, Desigualdades de Saúde, Pessoas com Deficiência, Autonomia Pessoal, Estudos de Prevalência

## Abstract

O objetivo deste estudo foi analisar fatores associados à dependência funcional (moderada e severa) entre pessoas idosas com deficiências sensoriais ou motoras no Brasil. Trata-se de estudo transversal com dados da *Pesquisa Nacional de Saúde* de 2019. A amostra incluiu 9.856 pessoas idosas (≥ 60 anos) com deficiência auditiva, visual ou motora. A variável de desfecho foi a dependência funcional, classificada em três níveis: independência, dependência funcional moderada e dependência funcional severa. Foram considerados fatores pessoais e fatores ambientais. Estimativas de prevalência e modelos de regressão logística multinomial, brutos e ajustados, foram estimados, adotando-se a independência como categoria de referência. Entre as pessoas idosas com deficiência, 44,4% viviam de forma independente, 33,6% apresentavam dependência moderada e 22% dependência severa. A dependência funcional (severa ou moderada) mostrou-se mais frequente entre mulheres, pessoas de maior idade e com menor escolaridade. A raça/cor apresentou associação apenas com a dependência funcional severa, com menores chances entre pessoas pardas em comparação às brancas. Entre os fatores ambientais, maiores chances de dependência foram observadas entre aqueles que não ocupavam posição de liderança no domicílio, residentes na Região Nordeste e usuários de tecnologias assistivas. A corresidência, o recebimento de visitas domiciliares de agentes comunitários de saúde e o uso de cuidados regulares de reabilitação estiveram associados especificamente à dependência funcional severa. A elevada proporção de pessoas idosas com deficiência que vivem de forma independente desafia concepções capacitistas sobre envelhecimento. Contudo, as desigualdades observadas indicam que a manutenção da autonomia funcional ainda é condicionada por fatores sociais e ambientais.

## Introdução

No paradigma do “envelhecimento bem-sucedido”, a autonomia e a independência são pilares consolidados na literatura como principais indicadores de bem-estar ^1^ ^,^ ^2^. Esses pilares surgem, dentre outras causas, com os objetivos de superar vieses excludentes e aproximar da realidade os paradigmas clássicos do envelhecimento. Inicialmente, porque o foco exacerbado na preservação das funções corporais e na evitação da deficiência, presentes nesses paradigmas clássicos, tendem a se tornar capacitistas ao atribuírem um menor valor às pessoas com deficiência ^2^. Pela outra parte, a ideia de que uma parcela significativa da população possa alcançar idades avançadas sem experienciar nenhuma deficiência não é sustentada pelos estudos populacionais ^1^ ^,^ ^2^.

No Brasil, segundo dados do *Censo Demográfico* de 2022 ^3^, 45% das pessoas com deficiência (aproximadamente 6,5 milhões) têm 60 anos ou mais, o que representa um quinto da população idosa do país. Entre os mais longevos, com 80 anos ou mais de idade, 40% vivem com alguma deficiência. Ademais, diante do contexto de envelhecimento populacional acelerado no país, é esperado que a parcela da população idosa com deficiência aumente ^4^. Nesse cenário, valorizar a velhice com independência, reconhecendo que uma boa vida é possível mesmo com deficiências, mostra-se um objetivo mais realista e menos capacitista para a sociedade brasileira do que a restrita priorização do envelhecer sem deficiência ^2^.

Elemento central para o bem-estar das pessoas idosas com deficiência, a autonomia, pode ser definida como a capacidade de manter um senso de agência pessoal, autoeficácia e poder de escolha ^2^. Wehmeyer ^5^, por sua vez destaca como elemento fundamental da autonomia, a autodeterminação, entendida como a capacidade dos indivíduos exercerem controle de suas próprias vidas. Sob essa perspectiva, a independência funcional, entendida como a ausência de necessidade de auxílio de terceiros para a realização de atividades cotidianas ^6^, constitui um importante aspecto a ser considerado na saúde dessa população.

Nem a independência funcional e nem a autodeterminação são uma questão de vontade/capacidade individual, mas sim um resultado do empoderamento das pessoas com deficiência ^5^. Empoderar, por sua vez, significa garantir os meios, o conhecimento e as oportunidades para que a autodeterminação seja possível.

Na perspectiva do empoderamento, o ambiente é a dimensão mais crucial, pois ele possibilita ou restringe a autonomia das pessoas com deficiência ^5^. Tal dimensão abrange aspectos tangíveis, como a infraestrutura física e serviços de reabilitação, e intangíveis, como as atitudes, culturas e crenças ^7^.

O principal referencial utilizado para operacionalizar os diferentes aspectos da funcionalidade, incluindo as interações entre os fatores ambientais e características individuais, é a *Classificação Internacional de Funcionalidade, Incapacidade e Saúde* (CIF) ^7^. Nesse modelo, a capacidade funcional refere-se à possibilidade de o indivíduo realizar atividades e participar da vida social, resultante da interação entre suas condições de saúde e o contexto em que vive. A independência funcional, por sua vez, é perdida a partir do momento que essa capacidade é reduzida a ponto de comprometer a autonomia para a realização de atividades cotidianas.

A relação entre o ambiente e as condições de vida da população com deficiência se reflete em desigualdades na prevalência de independência entre territórios e grupos sociais ^8^. Por sua vez, tais consequências ^7^ tendem a ser mais acentuadas na população idosa devido ao impacto cumulativo dos determinantes sociais da saúde ao longo da vida ^9^.

No Brasil, desigualdades sociais entre pessoas idosas têm sido evidenciadas quanto à independência funcional ^10^. Em geral, os grupos mais afetados são os mais longevos, mulheres e com menor nível socioeconômico. Os fatores associados à dependência funcional, no entanto, variam de acordo com o grau de dependência ^11^.

No entanto, os estudos sobre dependência em pessoas idosas no Brasil concentram-se na população idosa em geral ^6^ ^,^ ^10^ ^,^ ^12^. Assim, são desconhecidos os fatores associados à dependência da população idosa com deficiência, ainda que a autonomia seja um aspecto central para a compreensão do bem-estar de pessoas em condição de deficiência ^2^.

Duas hipóteses, fundamentadas com base no cenário exposto, nortearam o presente estudo: (1) que pessoas idosas com deficiência podem viver de forma independente no Brasil; (2) que determinantes pessoais e ambientais mediam as condições para uma vida independente nessa população, o que se manifestaria em desigualdades relativas à prevalência da dependência.

O objetivo do estudo foi analisar fatores associados à dependência funcional (moderada e severa) na população idosa com deficiência sensorial ou motora no Brasil.

## Métodos

### Desenho e fonte de dados

Trata-se de estudo transversal, analítico, de base populacional e com amostra representativa da população residente em domicílios particulares no Brasil ^13^. Utilizou-se como fonte de dados a *Pesquisa Nacional de Saúde* (PNS) de 2019 ^14^, realizada pelo Instituto Brasileiro de Geografia e Estatística (IBGE) entre agosto de 2019 e março de 2020.

A PNS é um inquérito de base domiciliar com delineamento amostral complexo. Na edição de 2019, contou com 94.114 domicílios com entrevista realizada. Para garantir a representatividade da amostra para a população brasileira foram aplicados fatores de expansão, incluindo um fator de correção para as perdas. Detalhes sobre o método de amostragem, fatores de ponderação e coleta de dados estão disponíveis em trabalhos anteriores ^13^ ^,^ ^14^.

O questionário da PNS 2019 foi dividido em três seções. As duas primeiras contêm dados sobre todos os moradores dos domicílios (n = 279.372), incluindo características da residência (seção 1), condições socioeconômicas, de acesso e uso de serviços de saúde, sobre deficiências, condições de saúde de pessoas com mais de 60 anos e menores de 2 anos (seção 2). A terceira seção contém informações sobre um morador de cada domicílio, selecionado aleatoriamente, e aborda questões relacionadas à saúde, estilo de vida, apoio social e características do trabalho.

Apenas as duas primeiras seções da PNS foram utilizadas no presente estudo, visando à inclusão de um maior número de pessoas na amostra.

### Amostra e variáveis

Os critérios de inclusão da amostra foram: ter 60 anos ou mais e apresentar algum tipo de deficiência. Foram excluídos indivíduos que se autodeclararam de raça/cor indígena (n = 128) ou amarela (n = 82), devido ao baixo número de casos, bem como aqueles com deficiência mental (n = 1.339), uma vez que a pergunta utilizada para identificar essa condição já incorpora o relato de dificuldade funcional nas atividades da vida diária, o que tornaria a análise tautológica. A amostra final foi constituída por 9.856 pessoas idosas com deficiência sensorial ou motora.

Foram consideradas as pessoas idosas com deficiência sensorial ou motora aquelas que, permanentemente, possuíam muita dificuldade ou não conseguiam de modo algum executar pelo menos uma das seguintes atividades: enxergar; ouvir; caminhar ou subir escadas; levantar uma garrafa com dois litros de água da cintura até a altura dos olhos; e pegar objetos pequenos, como botões e lápis, ou abrir e fechar recipientes ou garrafas.

A variável de desfecho foi a dependência funcional, considerada em três níveis distintos: (i) independência funcional; (ii) dependência funcional moderada; e (iii) dependência funcional severa.

Foram considerados com deficiência funcional severa todos aqueles que dependiam de ajuda para a realização de atividades básicas da vida diária (ABVD). Sua identificação foi feita a partir da reposta “sim” à pergunta “Precisa de ajuda para realizar algumas(s) destas atividades (comer, tomar banho, ir ao banheiro, se vestir, andar em casa de um cômodo ao outro, deitar-se ou levantar-se da cama sozinho, sentar-se ou levantar-se da cadeira sozinho)?”.

Foram considerados com dependência funcional moderada aqueles que, ao mesmo tempo, necessitavam de ajuda para a realização de atividades instrumentais da vida diária (AIVD) e não necessitavam de ajuda para ABVD. A necessidade de ajuda para a realização de AIVD foi identificada a partir da resposta “sim” à pergunta “Precisa de ajuda para realizar algumas(s) destas atividades (fazer compras, administrar as finanças, tomar os remédios, ir ao médico, sair utilizando um transporte ônibus, metrô, táxi, carro etc.)?”.

Pessoas que não possuíam dependência funcional moderada e severa foram consideradas independência funcional.

Partindo da compreensão da funcionalidade da CIF ^7^, o presente estudo incorporou como variáveis independentes:

(i) Fatores pessoais: (1) o sexo (masculino e feminino); (2) a faixa etária em anos (60-69; 70-79; e 80 ou mais); (3) a raça/cor da pele (branca, preta ou parda); (4) a escolaridade (Sem Instrução, Fundamental incompleto, Ensino Fundamental completo a Ensino Médio completo, Ensino Superior incompleto ou mais); (5) a renda domiciliar *per capita* em salários mínimos (até 1/2, mais de 1/2 até 1, mais de 1).

(ii) Fatores ambientais relacionados ao domicílio: (1) a corresidência da pessoas idosas com deficiência sensorial ou motora (mora sozinho, mora com uma pessoa, mora com duas pessoas ou mais); (2) a condição no domicílio (chefe do domicílio/cônjuge do chefe ou outra condição no domicílio); (3) a macrorregião de residência; (4) a adequação do domicílio, sendo considerado adequado o domicílio que tinha, ao mesmo tempo, densidade de moradores por dormitório ≥ 2, água canalizada em pelo menos um cômodo, escoamento de esgoto por rede geral de esgoto, rede pluvial ou fossa séptica e coleta de lixo por serviço de limpeza ^15^; (5) o acesso à internet no domicílio.

(iii) Fatores ambientais relacionados ao uso de tecnologias e serviços de saúde: (1) frequência de visitas domiciliares de agentes comunitários de saúde (ACS) nos últimos 12 meses; (2) uso de tecnologia assistiva para a sua deficiência, sendo considerados aqueles que faziam uso de tecnologia(s) assistiva(s) destinada(s) ao auxílio da função(ões) comprometida(s); (3) o recebimento de cuidados de reabilitação regular nos últimos 12 meses (tais como: fisioterapia, terapia ocupacional, fonoaudiologia, psicoterapia etc.).

### Análise estatística

A distribuição percentual das pessoas idosas com deficiência sensorial e motora pelos fatores ambientais, bem como a prevalência de cada um dos níveis de dependência, foram estimadas considerando-se o plano amostral da pesquisa, junto aos respectivos intervalos de 95% de confiança (IC95%).

Para investigar os fatores associados à dependência funcional em pessoas idosas com deficiência sensorial e motora, foram estimados modelos de regressão logística multinomial, sendo a variável dependente a dependência funcional, considerada em três categorias: independência (referência), moderada e severa. Inicialmente, realizaram-se análises brutas e, posteriormente, modelos ajustados, nos quais foram incluídas apenas as variáveis independentes que apresentaram associação estatisticamente significativa na análise bruta. Os resultados do modelo foram apresentados em *odds ratios* (OR), juntos aos seus respectivos intervalos de confiança e valores de p, adotando-se o nível de 5% de significância.

Todas as análises foram conduzidas por meio do pacote estatístico IBM SPSS Statistics, versão 21 (https://www.ibm.com/) - por meio do módulo de amostras complexas (*complex sample*).

O projeto da PNS 2019 foi aprovado pela Comissão Nacional de Ética em Pesquisa, Conselho Nacional de Saúde (CONEP/CNS, sob o parecer nº 3.529.376).

## Resultados

Em 2019, quase metade (44,4%; IC95%: 42,8-46,0) das pessoas idosas com deficiência sensorial ou motora residentes em domicílios particulares no Brasil eram independentes (Tabela 1), 33,6% (IC95%: 32,0-35,1) tinham dependência funcional moderada, e 22% (IC95%: 20,7-23,4) estavam em situação de dependência funcional severa.

**Tabela 1:** Grau de dependência funcional e características pessoais e ambientais da população idosa (com 60 anos ou mais) com deficiência sensorial ou motora. Brasil, 2019.

Variáveis	n	%	IC95%
Total	9.856	100,0	-
Dependência funcional			
Grau severo	2.170	22,0	20,7-23,4
Grau moderado	3.308	33,6	32,0-35,1
Independente	4.377	44,4	42,8-46,0
Fatores pessoais			
Sexo			
Masculino	3.435	34,9	33,5-36,2
Feminino	6.420	65,1	63,8-66,5
Faixa etária (anos)			
60-69	3.950	40,1	38,5-41,7
70-79	3.286	33,3	31,9-34,9
80 anos e mais	2.619	26,6	25,1-28,1
Raça/Cor da pele			
Branca	4.677	47,5	45,8-49,1
Preta	1.132	11,5	10,4-12,6
Parda	4.047	41,1	39,6-42,6
Renda domiciliar *per capita* em salários mínimos			
Até 1/2	1.777	18,0	16,8-19,3
1/2 até 1	4.501	45,7	44,0-47,4
Mais de 1	3.576	36,3	34,6-38,0
Escolaridade			
Sem instrução	2.654	26,9	25,5-28,4
Ensino Fundamental incompleto	5.047	51,2	49,6-52,8
Ensino Fundamental completo a Médio completo	1.654	16,8	15,6-18,0
Superior incompleto ou mais	500	5,1	4,4-5,8
Fatores ambientais			
Acesso à internet no domicílio			
Sim	5.722	58,1	56,3-59,8
Não	4.133	41,9	40,2-43,7
Corresidência			
Mora sozinho	1.949	19,8	18,5-21,1
Mora com uma pessoa	3.829	38,8	37,2-40,5
Mora com duas pessoas ou mais	4.077	41,4	39,8-43,0
Condição no domicílio			
Chefe ou cônjuge do chefe	8.148	82,7	81,4-83,8
Não é chefe e nem cônjuge do chefe	1.708	17,3	16,2-18,6
Mora em domicílio adequado *			
Não	2.816	28,6	27,0-30,2
Sim	7.040	71,4	69,8-73,0
Região de residência			
Norte	681	6,9	6,5-7,4
Nordeste	2.931	29,7	28,6-30,9
Sudeste	4.241	43,0	41,6-44,5
Sul	1.424	14,5	13,7-15,3
Centro-oeste	578	5,9	5,4-6,3
Frequência de visitas domiciliares de ACS			
Mensalmente	2.867	29,1	27,5-30,7
De 1 a 6 visitas	2.420	24,6	23,1-26,0
Nunca recebeu	4.569	46,4	44,5-48,2
Nos últimos 12 meses recebeu cuidados de reabilitação			
Sim	2.056	20,9	19,6-22,2
Não	7.800	79,1	77,8-80,4
Faz uso de tecnologia assistiva			
Não	5.972	60,6	59,1-62,1
Sim	3.883	39,4	37,9-40,9

ACS: agentes comunitários de saúde; IC95%: intervalo de 95% de confiança.

Fonte: *Pesquisa Nacional de Saúde* de 2019 ^14^.

* Domicílio adequado: até 2 moradores por dormitório, com água canalizada, esgotamento sanitário adequado e coleta de lixo.

Quanto às características das pessoas idosas com deficiência sensorial ou motora (Tabela 1), observou-se que mais de 60% eram do sexo feminino e tinham 70 anos ou mais de idade, e pouco mais da metade era negra (preta ou parda). A precariedade das condições de vida dessa população é notável: 78% não haviam concluído sequer o Ensino Fundamental e somente 37% tinham renda domiciliar *per capita* igual ou superior a 1 salário mínimo.

Quase 20% das pessoas idosas com deficiência sensorial ou motora moravam sozinhas e proporção semelhante não ocupava lugar de liderança no domicílio (Tabela 1). Cerca de 7 em cada 10 residiam nas regiões Sudeste ou Nordeste, fração próxima em domicílios adequados. Em contraste, o acesso à internet no domicílio foi menos frequente (42% não possuíam).

Percentuais elevados de pessoas idosas com deficiência sensorial ou motora não faziam uso de tecnologias assistivas para sua deficiência (61%) e não receberam visitas de ACS nos 12 meses anteriores à pesquisa (46%) (Tabela 1). Receberam cuidados regulares de reabilitação 21% da amostra no mesmo período. No entanto, vale ressaltar que é desconhecido o tipo de cuidado recebido, podendo esse não ser relacionado à reabilitação da função corporal comprometida.

A Tabela 2 apresenta o grau de dependência funcional nas pessoas idosas com deficiência sensorial ou motora, de acordo com as características pessoais e ambientais. Observa-se que a dependência funcional severa foi mais prevalente entre os seguintes grupos: que não estavam na condição de chefes/cônjuges do chefe do domicílio (43,9%; IC95%: 40,0-47,8); com 80 anos ou mais de idade (37,2%; IC95%: 34,3-40,2); que faziam uso de tecnologia assistiva (31,9%; IC95%: 29,5-34,5); e que receberam cuidados de reabilitação regular no último ano (31,6%; IC95%: 28,5-34,9). Os menos afetados, com prevalência de dependência funcional severa abaixo dos 15%, foram aqueles com 60-69 anos de idade, que residiam sozinhos e que possuíam Ensino Superior incompleto ou mais.

**Tabela 2:** Grau de dependência funcional em pessoas idosas com deficiência sensorial ou motora, segundo características pessoais e ambientais. Brasil, 2019.

Variáveis	Grau de dependência funcional para atividades da vida diária								
	Independente			Dependência moderada			Dependência severa		
	n	%	IC95%	n	%	IC95%	n	%	IC95%
Total	4.377	44,4	42,8-46,0	3.308	33,6	32,0-35,1	2.170	22,0	20,7-23,4
Fatores pessoais									
Sexo									
Masculino	1.791	52,1	49,5-54,7	906	26,4	24,2-28,7	738	21,5	19,3-23,9
Feminino	2.586	40,3	38,3-42,3	2.402	37,4	35,4-39,4	1.432	22,3	20,7-24,0
Faixa etária (anos)									
60-69	2.427	61,4	59,0-63,9	1.001	25,4	23,2-27,6	522	13,2	11,5-15,2
70-79	1.442	43,9	41,4-46,4	1.170	35,6	33,1-38,2	674	20,5	18,4-22,8
80 e mais	508	19,4	16,8-22,3	1.137	43,4	40,4-46,5	974	37,2	34,3-40,2
Raça/Cor da pele									
Parda	1.831	45,3	43,0-47,5	1.392	34,4	32,1-36,7	823	20,3	18,6-22,2
Preta	496	43,9	39,2-48,7	386	34,1	30,0-38,4	249	22,0	18,3-26,2
Branca	2.049	43,8	41,3-46,3	1.530	32,7	30,4-35,1	1.098	23,5	21,4-25,7
Escolaridade									
Superior incompleto ou mais	333	66,7	60,1-72,7	92	18,4	14,0-23,8	75	14,9	10,8-20,2
Ensino Fundamental completo a Médio completo	897	54,2	50,4-58,0	394	23,8	20,9-26,9	364	22,0	18,9-25,4
Ensino Fundamental incompleto	2.307	45,7	43,5-47,9	1.709	33,9	31,7-36,0	1.031	20,4	18,7-22,3
Sem instrução	840	31,6	29,0-34,5	1.114	42,0	39,1-44,9	700	26,4	23,9-29,1
Renda domiciliar *per capita* em salários mínimos									
Até 1/2	776	43,7	40,0-47,4	598	33,6	30,2-37,3	403	22,7	19,8-25,8
Mais de1/2 até 1	1.875	41,7	39,4-43,9	1.625	36,1	34,0-38,2	1.001	22,2	20,4-24,2
Mais de 1	1.725	48,2	45,6-50,9	1.085	30,3	27,7-33,1	766	21,4	19,3-23,8
Fatores ambientais									
Acesso à internet no domicílio									
Não tem acesso	1.722	41,7	39,3-44,1	1.551	37,5	35,2-39,9	860	20,8	18,8-23,0
Tem acesso	2.655	46,4	44,3-48,5	1.757	30,7	28,8-32,7	1.310	22,9	21,1-24,7
Condição no domicílio									
Chefe ou cônjuge do chefe	4.036	49,5	47,8-51,3	2.690	33,0	31,4-34,7	1.421	17,4	16,1-18,9
Outra condição	341	20,0	17,1-23,1	618	36,2	32,6-39,9	749	43,9	40,0-47,8
Corresidência									
Mora sozinho	945	48,5	44,7-52,3	751	38,5	35,0-42,2	253	13,0	10,8-15,5
Mora com uma pessoa	1.770	46,2	43,6-48,9	1.233	32,2	29,9-34,6	826	21,6	19,3-24,1
Mora com duas pessoas ou mais	1.662	40,8	38,4-43,1	1.323	32,5	30,2-34,8	1.092	26,8	24,8-28,9
Região de residência									
Norte	305	44,7	41,0-48,5	248	36,5	32,7-40,5	128	18,8	16,1-21,8
Sudeste	1.885	44,4	41,6-47,3	1.479	34,9	32,0-37,8	877	20,7	18,4-23,1
Centro-oeste	266	45,9	41,1-50,9	184	31,8	27,8-36,1	129	22,3	18,4-26,8
Sul	776	54,5	50,9-58,0	329	23,1	20,3-26,1	319	22,4	19,6-25,5
Nordeste	1.146	39,1	36,6-41,7	1.068	36,4	34,0-38,9	717	24,5	22,1-27,0
Domicílio adequado *									
Reside	3.222	45,8	43,8-47,8	2.306	32,8	30,9-34,7	1512	21,5	20,0-23,1
Não reside	1.155	41,0	38,5-43,6	1.002	35,6	33,0-38,3	658	23,4	20,8-26,2
Visitas domiciliares de ACS no ano									
Mensalmente	2.187	47,9	45,5-50,3	1.456	31,9	29,7-34,1	925	20,2	18,3-22,3
De 1 a 6 visitas	1.016	42,0	39,1-44,9	878	36,3	33,3-39,3	526	21,7	19,5-24,1
Não recebeu	1.174	40,9	37,9-44,1	974	34,0	31,0-37,1	719	25,1	22,6-27,7
Reabilitação regular no último ano									
Não recebeu cuidados	3.621	46,4	44,6-48,2	2.659	34,1	32,3-35,9	1.520	19,5	18,0-21,1
Recebeu cuidados	756	36,8	33,5-40,1	649	31,6	28,5-34,8	651	31,6	28,5-34,9
Tecnologia assistiva									
Não utiliza	2.964	49,6	47,6-51,7	2.079	34,8	32,9-36,7	930	15,6	14,2-17,1
Utiliza	1.414	36,4	33,9-39,0	1.230	31,7	29,2-34,2	1.240	31,9	29,5-34,5

ACS: agente comunitário de saúde; IC95%: intervalo de 95% de confinça.

Fonte: *Pesquisa Nacional de Saúde* de 2019 ^14^.

* Domicílio adequado: até 2 moradores por dormitório, com água canalizada, esgotamento sanitário adequado e coleta de lixo.

A dependência funcional moderada, por sua vez, foi mais prevalente nos seguintes grupos: com 80 anos ou mais (43,4%; IC95%: 40,4-46,5); sem instrução (42%; IC95%: 39,1-44,9); e que residiam em domicílios unipessoais (38,5%; IC95%: 35,0-42,2) (Tabela 2). Os destaques entre as menores prevalências (< 25%) foram observados entre pessoas idosas com deficiência sensorial ou motora com maiores níveis de escolaridade e residentes na Região Sul.

Por fim, quanto à independência funcional, observa-se maior prevalência entre as pessoas idosas com deficiência sensorial ou motora: menos longevas, com maior escolaridade, residentes da Região Sul, do sexo masculino e que não faziam uso de tecnologia assistiva (Tabela 2).

Os modelos de regressão logística que tiveram como desfecho a dependência funcional moderada e como categoria de referência as pessoas idosas com deficiência sensorial ou motora independentes revelaram que tanto fatores pessoais quanto contextuais influenciam as chances de apresentar esse nível de dependência (Tabela 3). Entre os fatores pessoais, a faixa etária e a escolaridade mostraram forte associação com a dependência funcional moderada na análise ajustada. A chance de dependência funcional moderada foi 4,45 vezes maior (IC95%: 3,50-5,64) entre pessoas com 80 anos ou mais, quando comparadas àquelas de 60-69 anos. Da mesma forma, indivíduos sem instrução apresentaram 3,33 vezes mais chances (IC95%: 2,22-5,01) em relação àqueles com Ensino Superior incompleto ou mais. O sexo feminino também esteve associado à maior prevalência de dependência funcional moderada, com 88% mais chances (IC95%: 1,60-2,21) em comparação aos homens. A renda domiciliar *per capita*, embora tenha apresentado associação na análise bruta, perdeu significância estatística após o ajuste pelos demais fatores incluídos no modelo.

**Tabela 3:** Análise bruta e ajustada dos fatores associados à dependência funcional moderada em pessoas idosas com deficiência sensorial ou motora. Brasil, 2019.

Variáveis	Dependência moderada					
	OR bruta			OR ajustada		
	OR	IC95%	Valor de p	OR	IC95%	Valor de p
Fatores pessoais						
Sexo						
Feminino	1,84	1,58-2,14	< 0.001	1,88	1,60-2,21	< 0,001
Faixa etária (anos)						
80 ou mais	5,42	4,35-6,77	< 0.001	4,45	3,50-5,64	< 0,001
70-79	1,97	1,67-2,32	< 0.001	1,80	1,52-2,14	< 0,001
60-69	1,00	-	-	1,00	-	-
Raça/Cor da pele						
Parda	1,02	0,87-1,20	0,824	-	-	-
Preta	1,04	0,82-1,32	0,733	-	-	-
Branca	1,00	-	-	-	-	-
Escolaridade						
Sem instrução	4,82	3,35-6,94	< 0,001	3,33	2,22-5,01	< 0,001
Ensino Fundamental incompleto	2,69	1,89-3,82	< 0,001	2,40	1,65-3,49	< 0,001
Ensino Fundamental completo a Médio completo	1,59	1,09-2,34	0,017	1,61	1,08-2,40	0,020
Superior incompleto ou mais	1,00	-	-	1,00	-	-
Renda domiciliar *per capita* em salários mínimos						
Até 1/2	1,23	0,98-1,53	0,075	1,14	0,89-1,46	0,305
Mais de 1/2 até 1	1,38	1,17-1,63	< 0,001	1,13	0,93-1,36	0,212
Mais de 1	1,00	-	-	1,00	-	-
Fatores ambientais						
Acesso à internet no domicílio						
Não possui	1,36	1,18-1,57	< 0.001	1,12	0,95-1,33	0,186
Corresidência						
Mora com 2 pessoas ou mais	1,00	0,82-1,22	0,987	-	-	-
Mora com 1 pessoa	0,88	0,72-1,07	0,199	-	-	-
Mora sozinho	1,00	-	-	-	-	-
Condição no domicílio						
Não é chefe e nem cônjuge do chefe do domicílio	2,72	2,19-3,38	< 0.001	1,96	1,55-2,48	< 0,001
Domicílio adequado *						
Não reside	1,21	1,04-1,41	0,012	1,07	0,90-1,26	0,456
Região de residência						
Norte	1,92	1,50-2,46	< 0.001	1,73	1,33-2,25	< 0,001
Nordeste	2,20	1,78-2,70	< 0.001	1,87	1,49-2,35	< 0,001
Sudeste	1,85	1,48-2,31	< 0.001	1,85	1,47-2,32	< 0,001
Centro-oeste	1,63	1,24-2,14	< 0.001	1,49	1,12-1,99	0,006
Sul	1,00	-	-	1,00	-	-
Visita domiciliar de ACS nos últimos 12 meses						
Recebeu pelo menos 1 visita	1,27	1,09-1,47	0,002	1,15	0,97-1,36	0,101
Cuidado de reabilitação regular nos últimos 12 meses						
Recebeu	1,17	0,97-1,41	0,094	-	-	-
Tecnologia assistiva						
Utiliza	1,24	1,06-1,45	0,007	1,27	1,08-1,49	0,004

ACS: agentes comunitários de saúde; IC95%: intervalo de 95% de confiança; OR: *odds ratio*.

Fonte: *Pesquisa Nacional de Saúde* de 2019 ^14^.

* Domicílio adequado: até 2 moradores por dormitório, com água canalizada, esgotamento sanitário adequado e coleta de lixo.

Entre os fatores contextuais que permaneceram associados à dependência funcional moderada na análise ajustada, destacaram-se a condição no domicílio, a região de residência e o uso de tecnologia assistiva (Tabela 3). Pessoas idosas que não eram chefes nem cônjuges do chefe do domicílio apresentaram 1,96 (IC95%: 1,55-2,48) vez mais chances de dependência funcional moderada, quando comparadas àquelas que ocupavam essas posições. Maiores chances entre residentes no Nordeste (OR = 1,87; IC95%: 1,49-2,35), Sudeste (OR = 1,85; IC95%: 1,47-2,32), Norte (OR = 1,73; IC95%: 1,33-2,25) e Centro-oeste (OR = 1,49; IC95%: 1,12-1,99) em comparação aos residentes da Região Sul, também foram observadas. Com menor intensidade, o uso de tecnologia assistiva também foi associado à dependência funcional moderada, sendo 27% (IC95%: 1,08-1,49) maiores as chances entre aqueles que relataram sua utilização.

Quanto aos modelos que apresentaram como desfecho a dependência funcional severa (Tabela 4), observou-se relativa semelhança entre os fatores associados com a dependência funcional moderada. Entre os fatores pessoais, a faixa etária apresentou a associação de maior magnitude, sendo 7,00 (IC95%: 5,39-9,10) vezes maiores as chances de dependência funcional severa entre pessoas com 80 anos ou mais, em comparação às pessoas de 60-69 anos. A escolaridade também demonstrou forte associação, com maiores chances entre indivíduos com menor nível educacional: pessoas sem instrução apresentaram 3,14 (IC95%: 1,93-5,12) vezes mais chances de dependência funcional severa, em comparação às que possuíam Ensino Superior incompleto ou mais. O sexo feminino também esteve associado à dependência funcional severa, com 27% mais chances de apresentar essa condição em relação aos homens (OR = 1,27; IC95%: 1,05-1,53). Diferentemente da análise do desfecho de dependência funcional moderada, quanto à raça/cor da pele observou-se que pessoas pardas apresentaram 22% (OR = 0,78; IC95%: 0,62-0,98) menores chances de dependência funcional severa quando comparadas às pessoas brancas. A renda domiciliar *per capita* não manteve associação estatisticamente significativa após o ajuste pelas demais variáveis incluídas no modelo, à semelhança do modelo relativo ao grau moderado de dependência.

**Tabela 4:** Análise bruta e ajustada dos fatores associados à dependência funcional severa em pessoas idosas com deficiência sensorial ou motora. Brasil, 2019.

Variáveis	Dependência severa					
	OR bruta			OR ajustada		
	OR	IC95%	Valor de p	OR	IC95%	Valor de p
Fatores pessoais						
Sexo						
Feminino	1,34	1,13-1,59	0,001	1,27	1,05-1,53	0,014
Faixa etária (anos)						
80 ou mais	8,91	7,00-11,34	< 0.001	7,00	5,39-9,10	< 0,001
70-79	2,17	1,75-2,71	< 0.001	2,12	1,67-2,68	< 0,001
60-69	1,00	-	-	1,00	-	-
Raça/Cor da pele						
Parda	0,84	0,71-1,00	0,044	0,78	0,62-0,98	0,034
Preta	0,94	0,70-1,25	0,662	0,93	0,66-1,31	0,68
Branca	1,00	-	-	1,00	-	-
Escolaridade						
Sem instrução	3,73	2,47-5,62	< 0,001	3,14	1,93-5,12	< 0,001
Ensino Fundamental incompleto	2,00	1,35-2,96	0,001	2,21	1,42-3,44	< 0,001
Ensino Fundamental completo a Médio completo	1,81	1,19-2,77	0,006	2,20	1,37-3,53	0,001
Superior incompleto ou mais	1,00	-	-	1,00	-	-
Renda domiciliar *per capita* em salários mínimos						
Até 1/2	1,17	0,93-1,47	0,181	1,36	1,00-1,84	0,052
Mais de 1/2 até 1	1,20	1,01-1,43	0,037	1,24	1,00-1,55	0,051
Mais de 1	1,00	-	-	1,00	-	-
Fatores ambientais						
Acesso à internet no domicílio						
Não possui	1,01	0,85-1,21	0,888	-	-	-
Corresidência						
Mora com 2 pessoas ou mais	2,46	1,92-3,14	< 0.001	1,48	1,13-1,93	0,005
Mora com 1 pessoa	1,75	1,33-2,29	< 0.001	1,60	1,20-2,14	0,001
Mora sozinho	1,00	-	-	1,00	-	-
Condição no domicílio						
Não é chefe e nem cônjuge do chefe do domicílio	6,24	4,97-7,83	< 0.001	3,92	3,01-5,11	< 0,001
Domicílio adequado *						
Não reside	1,21	1,01-1,46	0,042	1,13	0,91-1,41	0,276
Região de residência						
Norte	1,02	0,78-1,34	0,887	1,20	0,87-1,65	0,279
Nordeste	1,52	1,20-1,92	< 0.001	1,56	1,16-2,10	0,004
Sudeste	1,13	0,89-1,43	0,307	1,19	0,90-1,57	0,22
Centro-oeste	1,18	0,85-1,63	0,319	1,18	0,83-1,68	0,371
Sul	1,00	-	-	1,00	-	-
Visita domiciliar de ACS nos últimos 12 meses						
Recebeu pelo menos 1 visita	1,34	1,14-1,58	< 0,001	1,27	1,04-1,54	0,019
Cuidado de reabilitação regular nos últimos 12 meses						
Recebeu	2,05	1,68-2,50	< 0.001	2,85	2,26-3,60	< 0,001
Tecnologia assistiva						
Utiliza	2,80	2,34-3,33	< 0.001	2,76	2,27-3,36	< 0,001

ACS: agentes comunitários de saúde; IC95%: intervalo de 95% de confiança; OR: *odds ratio*.

Fonte: *Pesquisa Nacional de Saúde* de 2019 ^14^.

* Domicílio adequado: até 2 moradores por dormitório, com água canalizada, esgotamento sanitário adequado e coleta de lixo.

Com relação aos fatores ambientais, a análise ajustada evidenciou associação significativa entre a dependência funcional severa e as variáveis relacionadas aos arranjos domiciliares, à região de residência e ao acesso a serviços e recursos de saúde (Tabela 4). A condição no domicílio apresentou a associação de maior magnitude, sendo 3,92 vezes maiores as chances de dependência funcional severa entre pessoas idosas com deficiência sensorial ou motora que não eram chefes nem cônjuges do chefe do domicílio (IC95%: 3,01-5,11). Em relação à corresidência, observou-se maior probabilidade de dependência funcional severa entre aqueles que moravam com duas pessoas ou mais (OR = 1,48; IC95%: 1,13-1,93) ou com apenas uma pessoa (OR = 1,60; IC95%: 1,20-2,14), quando comparados aos que moravam sozinhos. A região de residência também apresentou associação significativa, com 56% mais chances de dependência funcional severa entre os residentes na Região Nordeste (OR = 1,56; IC95%: 1,16-2,10), em comparação aos residentes na Região Sul.

Pessoas com acesso a serviços e tecnologias de saúde apresentaram maiores chances de ter dependência funcional severa (Tabela 4). O recebimento de visita domiciliar de ACS esteve associado a 27% mais chances de dependência funcional severa (OR = 1,27; IC95%: 1,04-1,54). Não obstante, associações mais fortes foram observadas quanto ao recebimento de cuidados de reabilitações regulares (OR = 2,85; IC95%: 2,26-3,60) e o uso de tecnologia assistiva (OR = 2,76; IC95%: 2,27-3,36), demonstrando maiores chances de dependência funcional severa entre aqueles que relataram utilizar esses recursos.

## Discussão

Um primeiro achado que merece destaque no presente estudo, sobretudo pela sua capacidade de desmitificar vieses capacitistas presentes em diversos paradigmas sobre saúde e envelhecimento, refere-se à observação de que quase metade das pessoas idosas com deficiência sensorial ou motora no Brasil tinham vidas funcionalmente independentes. Embora seja uma estimativa descritiva e simples, ela, por si só, confirma a primeira hipótese do estudo, de que a vida com deficiência pode se dar de forma independente, mesmo na população idosa.

Entretanto, as possibilidades de experimentar essa condição de independência não se apresentam de maneira equitativa, conforme evidenciado pela análise dos fatores associados à dependência funcional. As desigualdades observadas entre os grupos analisados sugerem que, tanto barreiras (materiais e imateriais) socialmente construídas quanto fatores biológicos influenciam as condições para a vida independente nessa população. A dependência funcional foi mais frequente entre pessoas do sexo feminino, mais longevas e com menor escolaridade, evidenciando importantes desigualdades sociodemográficas. No que se refere ao contexto ambiental, observou-se que as chances de dependência funcional, tanto moderada quanto severa, foram maiores entre pessoas idosas com deficiência sensorial ou motora que não ocupavam posição de liderança no domicílio, que residiam na Região Nordeste e que faziam uso de tecnologias assistivas. Especificamente para a dependência funcional severa, verificou-se maior probabilidade entre aqueles que não residiam sozinhos, bem como entre os que receberam visita domiciliar de agente comunitário de saúde e cuidados regulares de reabilitação.

As características particulares dos fatores associados a cada grau de dependência na população de pessoas idosas com deficiência sensorial ou motora estão de acordo com os estudos para a população idosa em geral ^11^. Contudo, alguns dos aspectos em comum entre ambos os desfechos na presente pesquisa foram os observados quanto à renda domiciliar *per capita*, ao acesso à internet e à adequação do domicílio que não se associaram significativamente a nenhum deles nos modelos ajustados.

O sexo feminino apresentou desvantagens quanto à prevalência dependência funcional, especialmente no grau moderado. Esses achados estão em discordância com estudos de abrangência nacional ^12^ e locais ^11^ ^,^ ^16^, voltados para a população idosa em geral. No primeiro caso ^12^, o comprometimento para a realização das AIVD foi associado ao sexo feminino, no entanto, com menor intensidade do que a encontrada no presente estudo, com chances 26% maiores do que as do sexo masculino. Por outra parte, não observou-se associação para as ABVD ^12^. Tais diferenças indicam que as desvantagens femininas relativas à funcionalidade na faixa etária idosa são acentuadas quando em condição de deficiência. Nos estudos locais, o sexo não foi associado a nenhum dos desfechos ^11^ ^,^ ^16^. Fatores biológicos e relacionados à maior prevalência de doenças incapacitantes entre pessoas do sexo feminino ^17^ são os mais mencionados para a explicação das desvantagens femininas em termos de funcionalidade na população idosa ^16^.

A progressão das faixas etárias mostrou-se o principal fator associado à perda de independência em pessoas idosas com deficiência sensorial ou motora. Embora tenha sido fortemente associado a ambos os graus de dependência, ela foi destacadamente marcante para a dependência funcional severa, com chances expressivamente maiores entre aqueles que ultrapassaram os 80 anos. A associação entre idade e dependência, marcada por processos biológicos que geram um declínio da funcionalidade e são intrínsecos ao envelhecimento individual, também foi observada em estudos com pessoas idosas em geral ^11^ ^,^ ^12^ ^,^ ^16^. Nesses estudos, no entanto, a associação entre incapacidade funcional e faixa etária foi menos intensa do que a encontrada no presente estudo, além de apresentar-se mais forte para as AIVD do que para as ABVD ^11^ ^,^ ^16^. Tais diferenças indicam que a manutenção da independência para pessoas idosas com deficiência a partir dos 80 anos de idade torna-se uma condição particularmente desafiadora.

A raça/cor da pele não apresentou associação significativa com a dependência funcional moderada entre pessoas idosas com deficiência sensorial ou motora, resultado coerente com estudo voltado à população idosa em geral no Brasil ^12^. No entanto, indivíduos pardos apresentaram 22% menores chances de dependência funcional severa quando comparados aos brancos. A heterogeneidade social, cultural e fenotípica que caracteriza a categoria parda no contexto brasileiro dificulta a formulação de interpretações ^18^ sobre os possíveis fatores que poderiam explicar tal diferença.

A desigualdade socioeconômica no grau de dependência de pessoas idosas com deficiência sensorial ou motora no Brasil foi observada verificando-se a escolaridade. Tanto no grau moderado quanto no severo observaram-se desvantagens para aqueles com menor nível educacional. Considerando-se que a própria condição de deficiência entre pessoas idosas no Brasil é fortemente associada ao nível de escolaridade, sendo 2,23 vezes mais prevalentes entre pessoas idosas sem instrução se comparadas àquelas com Ensino Superior incompleto ou mais ^4^, essa realidade revela-se ainda mais problemática. Isso significa uma dupla carga de desvantagens das pessoas idosas menos escolarizadas: adquirem deficiências com maior frequência e, quando nessa condição, têm maiores chances de perderem sua independência.

A relação entre funcionalidade e escolaridade deve-se a uma multiplicidade de aspectos que mediam a relação entre o estado de saúde e o grau de instrução formal ^19^. Um aspecto mencionado na literatura, diz respeito à maior compreensão e adoção de hábitos saudáveis por pessoas com maior escolaridade, o que contribui para a preservação da capacidade funcional desses indivíduos ^20^. Nesse sentido, entre as pessoas idosas com deficiência sensorial ou motora, a prática de atividades físicas ao longo da vida ^21^ tem destacada importância em função do desenvolvimento e preservação de massa muscular, o que previne a sarcopenia ^22^. Muitas das vezes tal condição é a causa do desenvolvimento e do agravamento de deficiências motoras - tipo de deficiência mais prevalente na população idosa ^4^.

O menor acesso e o pior uso dos serviços e tecnologias de saúde por parte da população com menor escolaridade também são questões frequentemente mencionadas ^21^. O menor grau de instrução sujeita indivíduos a um menor conhecimento, aceitabilidade e compreensão de técnicas e tecnologias de saúde de manejo e reabilitação funcional, capazes de prevenir ou postergar a condição de dependência em função de sua deficiência. Por outro lado, a discriminação interpessoal e institucional, fruto da tripla estigmatização dessas pessoas como: (i) idosas, (ii) não alfabetizadas, e (iii) portadoras de deficiência ^23^, também podem afetar a acessibilidade desses indivíduos a tais técnicas e tecnologias.

As piores ocupações ao longo do curso da vida ^24^ também podem explicar a menor prevalência de independência entre pessoas idosas com deficiência sensorial ou motora com baixa escolaridade. Pessoas idosas e com menor escolaridade, com maior frequência trabalharam em ocupações degradantes que deterioraram por anos suas condições físicas ^25^.

Por fim, cabe destacar que a associação entre escolaridade e dependência funcional moderada foi levemente mais intensa do que com a dependência funcional severa, o que está de acordo com estudos voltados à população idosa em geral ^12^ ^,^ ^16^. Como as AIVD envolvem maior complexidade, é esperado que a necessidade de auxílio para realizá-las entre pessoas de menor escolaridade seja mais frequente do que para as tarefas básicas. Nos casos extremos, porém frequentes na população idosa, a condição de analfabetismo inviabiliza ou provoca séria dificuldade para a realização independente de atividades como ir ao médico, fazer compras, administrar finanças e tomar remédios ^16^.

Diferentemente da escolaridade, a renda domiciliar não foi associada à dependência, resultado consistente com o estudo realizado em Montes Claros (Minas Gerais) ^26^. Uma possível hipótese para esse achado é a de que as condições materiais para acessar serviços e bens que preservam ou manejam a funcionalidade não têm tanta influência sobre a condição independência entre pessoas idosas com deficiência sensorial ou motora no Brasil. Outro aspecto possível para a explicação desse achado decorre do êxito das políticas da Assistência Social e Previdência Social, que reduziram desigualdades e tornaram residual o nível de pobreza na população idosa ^27^.

No que diz respeito à análise dos fatores ambientais associados à dependência funcional, vale mencionar que em muitos casos esses ocorreram de forma inesperada. O acesso à internet no domicílio, que poderia facilitar a realização de AIVD ^28^, é um exemplo. Nesse caso, uma hipótese possível é a de que, mesmo com o acesso, o uso da internet pelas pessoas idosas com deficiência sensorial ou motora ainda seja limitado e pouco proveitoso.

Outro exemplo foi a adequação do domicílio. Por incluir como critério de adequação o acesso ao esgotamento sanitário e à água encanada ^15^, esperava-se que esse indicador apresentasse associação com a independência para realização de ABVD por parte das pessoas idosas com deficiência sensorial ou motora, sobretudo daquelas relacionadas à higiene. A estrutura da moradia é fundamental para a vida independente de pessoas com deficiência ^7^. Um exemplo simples é o de alguém com deficiência nos membros inferiores que vive em um domicílio onde o uso de escadas é necessário. Nesse caso, torna-se inevitável a dependência de auxílio para sair de casa. No entanto, as perguntas voltadas ao domicílio na PNS não contemplam características sobre a adaptabilidade para pessoas com deficiência, o que impossibilitou a adoção de um critério mais apropriado de adequação domiciliar.

O recebimento de cuidados de reabilitação e o uso de tecnologias assistivas também apresentaram resultados inesperados. Partindo dos pressupostos de que: (i) toda a amostra, em decorrência de suas deficiências, demandou cuidados de reabilitação e o uso de tecnologias assistivas; (ii) o uso desses recursos tem o potencial de preservar a autonomia ^29^, era esperado que a prevalência dependência fosse menor entre os usuários.

Esses achados são reflexos da limitação da natureza transversal da pesquisa, que não permite estabelecer relações de causa e efeito. O mais provável é que a busca por reabilitação e tecnologias assistivas esteja sendo gerada pelo grau severo da incapacidade funcional ^30^. Inferências mais sólidas sobre o pressuposto “o uso desses recursos tem o potencial de preservar a autonomia”, mencionado no parágrafo anterior, exigem, ao menos, estudos longitudinais com pessoas idosas com deficiência sensorial ou motora independentes.

Quanto ao menor uso desses recursos por pessoas idosas com deficiência sensorial ou motora com independência funcional ou dependência funcional severa, duas hipóteses são possíveis: (i) baixa aceitabilidade de pessoas com maior capacidade funcional às tecnologias assistivas devido ao estigma ligado ao seu uso ^31^; (ii) barreiras de acesso, caso a oferta pública esteja sendo insuficiente e priorizada para indivíduos em pior situação. Nesse cenário, é válido o questionamento de se a utilização tardia de serviços de reabilitação e tecnologias assistivas por pessoas idosas com deficiência sensorial ou motora com independência funcional ou com dependência funcional moderada não estaria gerando casos evitáveis ou precoces de dependência funcional severa.

A incerteza quanto à relação de causa e efeito também se aplica às associações com as visitas domiciliares de ACS. Embora seja esperado que a assistência domiciliar favoreça o acompanhamento, preservação e reabilitação da funcionalidade de pessoas idosas com deficiência sensorial ou motora ^32^, a associação encontrada indica que há uma priorização daquelas com dependência funcional severa no âmbito da atenção primária à saúde (APS). Por conta de suas dificuldades de locomoção aos estabelecimentos de saúde, tal priorização é, em parte, justificada.

Em contraposição, as associações relacionadas ao ambiente familiar apresentaram-se dentro do esperado. Ainda assim, as associações também parecem ser causadas pela condição de dependência, ao passo que a dependência condiciona as pessoas idosas com deficiência sensorial ou motora a um rearranjo familiar, não sendo, portanto, uma condição gerada pelo ambiente familiar. Diante da necessidade de auxílio para atividades básicas cotidianas, a corresidência com a pessoa idosa dependente torna-se um facilitador para a vida dos familiares cuidadores ^33^. Ao mesmo tempo, a partir do momento em que a pessoas idosas com deficiência sensorial ou motora passa a depender de cuidados, ela tende a perder a posição de liderança no ambiente domiciliar ^34^.

Finalmente, uma das desigualdades mais relevantes em termos de necessidade de atenção pública foi a relacionada à região de residência. Residentes nas regiões Nordeste, Sudeste, Norte e Centro-oeste, tiveram maiores chances de dependência funcional moderada em relação à Região Sul. A Região Nordeste, além de maior desvantagem para o grau moderado, também foi a única associada à dependência funcional severa. Pesquisa como a PNS 2013 encontrou cenário semelhante na população idosa em geral, com desvantagens mais acentuadas no Nordeste ^35^. O fato de a desigualdade regional se concentrar principalmente no grau moderado de dependência é compreensível, já que as AIVD estão mais relacionadas ao ambiente extradomiciliar. Assim, é plausível inferir que a Região Sul ofereça melhores condições para a vida independente das pessoas idosas co deficiência sensorial ou motora. Estudos que aprofundem os determinantes dessa desigualdade seriam valiosos para a orientação de políticas voltadas à superação dessas desigualdades regionais, fomentando políticas urbanas de adequação dos espaços e serviços públicos e privados nos territórios menos inclusivos.

Outras políticas, voltadas à promoção da independência de pessoas com deficiência e à redução das desigualdades observadas, devem contemplar: (i) o fortalecimento da APS, com a ampliação e a qualificação da oferta de cuidados de reabilitação e de ações de promoção da saúde, de modo a contribuir para a preservação da capacidade funcional de pessoas com deficiência ^36^; (ii) o fornecimento de tecnologias assistivas adequadas para o manejo da funcionalidade em pessoas com deficiência, associado ao acompanhamento e orientação que garantam o uso apropriado ^20^; e (iii) a adaptação dos domicílios às necessidades específicas de cada indivíduo, favorecendo maior autonomia e segurança no cotidiano ^7^.

O contexto da pandemia de COVID-19 evidenciou a urgência e a magnitude do problema dos cuidados destinados às pessoas idosas com dependência. Nos momentos mais críticos da crise sanitária, muitas dessas pessoas ficaram desprovidas de cuidados básicos e, quando os recebiam, muitas vezes eram de familiares sobrecarregados ^37^. Diante da expectativa de aumento de situações de crise sanitária associadas às mudanças climáticas ^38^, torna-se fundamental que a sociedade se prepare para esse cenário por meio de ações em múltiplas frentes, entre as quais a prevenção e o retardamento da dependência funcional.

Algumas limitações do estudo, como a natureza transversal da pesquisa, já foram discutidas ao longo do trabalho. Outra limitação que vale menção é o possível viés de sobrevivência ^39^. Pessoas idosas com deficiência sensorial ou motora com piores condições de vida podem nem chegar à condição de dependência ou sobreviver por pouco tempo quando a atingem. Nesse sentido, a magnitude das desigualdades observadas tende a ser subdimensionada frente à gravidade real.

Por outro lado, este estudo tem importantes fortalezas. Trata-se de investigação inédita em nível nacional, com amostra representativa, centrada em um grupo pouco estudado, embora numeroso e que aborda uma lacuna crítica nas informações sobre pessoas com deficiência.

O conhecimento sobre a condição de dependência de cuidados de pessoas com deficiência é especialmente relevante para as emergentes políticas de Cuidados. Marco fundamental dessa agenda, a recém-aprovada Política Nacional de Cuidados (*Lei nº 15.069/2024*) ^40^ define como um de seus públicos prioritários as “*pessoas com deficiência que necessitam de assistência, apoio ou auxílio para executar as atividades básicas e instrumentais da vida diária*”. Atualmente, o perfil e o tamanho desse público não são passíveis de serem conhecidos no nível nacional em populações não idosas. Nesse contexto, enfatiza-se a urgência da inclusão de perguntas sobre a necessidade de auxílio para a realização de ABVD e AIVD para todas as pessoas com deficiência em inquéritos futuros.

## Data Availability

As fontes de informação utilizadas no estudo estão indicadas no corpo do artigo.
